# MAP9 Exhibits Protumor Activities and Immune Escape toward Bladder Cancer by Mediating TGF-*β*1 Pathway

**DOI:** 10.1155/2022/3778623

**Published:** 2022-05-24

**Authors:** Chong Zhang, Bing Han, Yuanyuan Guo, Han Guan, Zhijun Chen, Beibei Liu, Wenyan Sun, Wenyong Li, Wei Sun, Sheng Wang

**Affiliations:** Department of Urinary Surgery, The First Affiliated Hospital of Bengbu Medical College, Bengbu, Anhui, China

## Abstract

To investigate more potential targets for the treatment of human bladder cancer, quantitative reverse transcription polymerase chain reaction (qRT-PCR) and high-content screening (HCS) analysis were performed, and microtubule-associated protein 9 (MAP9), which had the strongest proliferation inhibition from 809 downregulated genes, has been selected. MAP9 is responsible for bipolar spindle assembly and is involved in the progression of many types of tumors; however, its role in bladder cancer (BC) remains unknown. Expressive levels of MAP9 in BC tissues were determined through immunohistochemistry, and the clinical significance of MAP9 in BC was analyzed. Short hairpin ribonucleic acid- (ShRNA-) MAP9 was used to construct stable MAP9 knockdown BC cell lines. The proliferative abilities of MAP9 were measured through assays *in vivo* and *in vitro*, and the migrated and invasive abilities of MAP9 were analyzed via *in vitro* experiments. Quantitative reverse transcription PCR, western blotting, coimmunoprecipitation (Co-IP), and rescue assays were used to identify downstream targets of MAP9. MAP9 expression increased in the tumor tissues, and its increased level was negatively correlated with prognosis. Further, the loss of MAP9 caused decreased BC cell proliferation via inducing the growth 1/synthesis (G1/S) cell cycle arrest *in vitro* and slowed tumor growth *in vivo*. In addition, MAP9 silencing attenuated BC cell migration and invasion. Moreover, we found that the growth 1/synthesis (G1/S) cell cycle-related genes and the epithelial mesenchymal transition (EMT) marker levels decreased after silencing MAP9. Finally, we found that the transforming growth factor beta 1 (TGF-*β*1) pathway is activated as a mediator for MAP9 to regulate genes related to the G1/S cell cycle and EMT. MAP9 promotes BC progression and immune escape activity through the TGF-*β*1 pathway and is a potential novel target for therapies of BC.

## 1. Introduction

Bladder cancer (BC), with more than 357,000 new cases and more than 130,000 deaths per year worldwide, is one of the major causes of mortality in men. In Europe, 151,297 new cases of BC were diagnosed in 2012, with an incidence rate of 17.7/100,000 for men and 3.5/100,000 for women (per 100,000 people). Although comprehensive therapeutic methods, including operation, chemotherapy, and radiotherapy, have been employed for the treatment of BC, 52,395 BC patients died in 2012, with an annual mortality rate of 7.1/100,000 [1]. Biological therapies targeting oncogenes or tumor inhibitors bring hope for the tumor treatment. However, limited targets with clinical effects have been identified in BC. These highlight the urgency and importance of exploring targets for the biological therapy for BC.

Based on our previous report that the centromere protein U (CENPU) can be used as a potential target for the treatment of human BC, we hope to further investigate more potential targets for the treatment of human BC. Therefore, based on our previously reported gene chip results and the gene expression profile analysis results after CENPU knockout, a total of 1274 differentially expressed genes were found, including 809 downregulated genes and 465 upregulated genes [2]. Furthermore, we selected 33 from 809 downregulated genes, which are relatively few reported BC genes. Twenty-one candidate genes have been screened through quantitative reverse transcription polymerase chain reaction (qRT-PCR) quantification with consistent expression level at the cell and array levels. The 21 candidate genes' high-content screening (HCS) analysis revealed that knocking down MAP9 and MRPS28 has shown significant proliferation inhibition. MAP9, with the strongest proliferation inhibition at a 3.32-fold change, was selected as the target for follow-up research.

MAP9, also named as ASter-Associated Protein (ASAP), is a microtubule-associated protein that is responsible for bipolar spindle assembly, cytokinesis, and centrosome integrity [3, 4]. MAP9 is essential for development, and the loss of MAP9 in zebrafish embryo results in developmental defects, which eventually cause early embryonic lethality [5]. MAP9 modulates the mitotic spindle. In detail, the knockdown of MAP9 results in multipolar spindles and defects in cytokinesis, whereas overexpression leads to monopolar spindles [3]. These findings demonstrate that MAP9 is of great importance in mitotic progression. Thus, we hypothesized that MAP9 regulates the progression of tumors based on the above-mentioned evidence. In fact, the dysregulation or hypermethylation of MAP9 has been detected in certain types of tumors, such as hepatocellular carcinoma (HCC) and colorectal cancer [6, 7]. Moreover, MAP9 inhibits cell proliferation, colony formation, migration, and invasion of HCC cells [8]. Although the expression and function of MAP9 have been partly discovered in several cancers, its role in BC remains unclear.

To unveil the role of MAP9 in BC, we aimed to analyze the expression and clinical significance of MAP9 in BC samples and to investigate the biological function and mechanism of MAP9 in BC cell lines (T24 and 5637) and nude mice. Then, we aimed to explore the potential downstream targets of MAP9 in BC cells.

## 2. Materials and Methods

### 2.1. Celigo Select (HCS)

The target cells were cultivated in good growth condition, the cells were divided into 96-well culture plates, and then, the lentivirus infection experiment of the target cells was carried out. After infection for 2~3 d, the green fluorescent protein (GFP) expression of the cells with 70~90% fluorescence rate was observed, and the cells were collected. From the second day after plating, Celigo detection was performed, and the plate was read once daily. Then, the plate was continuously tested and read for 5 d. By adjusting the input parameters of the analysis settings, the number of cells with green fluorescence in each scan of the well was calculated. The data was statistically drawn to plot a proliferation curve of the cells over 5 d. The cell proliferation fold of each group was calculated, that is, the fold change value relative to the negative control (NC) group of the proliferation fold. The calculation formula used is shown in the following equation:

(1) $$\begin{array}{c} \text{fold change}=\frac{\text{cell count fold value of NC group}}{\text{cell count fold value of test group}}, \end{array}$$

wherein a fold change ≥ 2 means that the cell proliferation of the experimental group is significantly slower than that of the NC group.

### 2.2. Tissue Samples

Fifty paired cancerous and paracancerous tissues were obtained from patients who received surgery from the First Affiliated Hospital of Bengbu Medical University. None of these patients received radiotherapy or chemotherapy before the operation or experienced lymph node or distant metastasis. This study was approved by the local ethics committee of the First Affiliated Hospital of Bengbu Medical College.

### 2.3. Cell Culture

Two human BC cell lines (T24 and 5637) and 293T cells were purchased from the Cell Bank of Chinese Academy of Sciences (Shanghai, China) and maintained in Dulbecco's Modified Eagle Medium (DMEM; Gibco, Carlsbad, CA, USA) supplemented with 10% fetal bovine serum (FBS; Gibco, Carlsbad, CA, USA) and penicillin/streptomycin at 37°C (5% carbon dioxide [CO_2_]).

### 2.4. Quantitative Reverse Transcription Polymerase Chain Reaction

The total RNA of the T24 and 5637 cells was extracted via TRIzol Reagent (Invitrogen, Carlsbad, CA, USA) and was reversed to complementary deoxyribonucleic acid (cDNA) using a PrimeScript RT Reagent Kit (Promega, USA). The qRT-PCR was performed using SYBR Green (Takara, Japan). All experiments were carried out based on the manufacturer's protocol. The cycle time (Ct) values of the genes detected were normalized to glyceraldehyde 3-phosphate dehydrogenase (GAPDH). The primers for the selected genes are listed in Table S1.

### 2.5. Immunohistochemistry (IHC)

Immunohistochemistry staining was performed using DAB Horseradish Peroxidase Color Development Kit (Solarbio, Beijing, China). Four-micrometer paraffin-embedded tissue sections were deparaffinized and rehydrated in xylene and alcohol, respectively. Sections were heated for 5 min to repair antigenicity and then treated for 10 min with 3% hydrogen peroxide (H_2_O_2_) to inactivate endogenous peroxidase activity. Sections were incubated with the primary antibody overnight and with secondary antibody for 1 h in turn. DAB chromogenic agent and hematoxylin were used to stain the slices successively, and then, the slices were dehydrated, transparent, and sealed for observation with an optical microscope.

### 2.6. Western Blotting

The total protein was extracted from the T24 and 5637 cells by radioimmunoprecipitation assay (RIPA) buffer and quantified by bicinchoninic acid (BCA) Protein Assay Kit (Beyotime, Shanghai, China). Proteins were transferred to polyvinylidene difluoride (PVDF) membranes after being resolved with sodium dodecyl sulfate-polyacrylamide gel electrophoresis (SDS-PAGE). Membranes were incubated with primary antibodies at 4°C overnight after being blocked for 2 h. Secondary antibodies were then added to incubate for 2 h at 37°C. Proteins were visualized by the enhanced chemiluminescence (ECL) detection kit (Thermo Fisher, Waltham, MA, USA). The antibodies for the selected proteins are presented in Table S2. The PVDF membrane was then exposed using the Gel Imager System (Bio-Rad, USA). ImageJ software was used to analyze the relative expression of proteins.

### 2.7. Enzyme-Linked Immunosorbent Assay (ELISA)

The samples, antibody conjugate, horseradish peroxidase- (HRP-) streptavidin solution, and substrate solution were consecutively added into each well, and the well was incubated at 37°C for 15 min to 1.5 h according to the protocol provided. During each step, the wells were aspirated and washed with a wash buffer. Finally, stop solution was added, and the optical density (OD) value was read at 450 nm within 3 min.

### 2.8. Short Hairpin Ribonucleic Acid- (ShRNA-) MAP9 Stable MAP9 Silencing of BC Cells

Short hairpin RNA targeting MAP9 was established (Gene, Shanghai, China). The target sequence of sh-MAP9 is 5′-CTTTCCAGGATGAGCTAATAA-3′, and the designed sequences of sh-MAP9 and sh-ctrl (control group) were 5′-CCGGCTTTCCAG GATGAGCTAATAACTCGAGTTATTAGCTCATCCTGGAAAGTTTTTG-3′ and 5′-CCGGTGCCCGACTATATAGATATAACTCGAGTTATATCTATATAGTCGGGCATTTTTG-3′, respectively. Lentivirus was then infected into the T24 and 5637 cells for 48 h. Then, polybrene (3 *μ*g/mL) was added into the medium to determine the proper titer of the lentivirus, and 1*E*+8 TU/mL was selected. Finally, ShRNA-MAP9 stable MAP9 knockdown BC cells were filtered using puromycin (5 *μ*g/mL).

### 2.9. Cell Counting Kit-8 (CCK-8) Assay

Cell Counting Kit-8 assay was performed using a cell counting kit (Biolite, Xian, China) according to the manufacturer's protocol. The T24 and 5637 cells were seeded into 96-well plates at a density of 2 × 10^3^ cells/well with 200 *μ*L of culture medium. Twenty microliters of CCK-8 reagent was added into each well after culturing for 24 h. The absorbance at 450 nm wavelength was detected at different time points (24, 48, 72, 96, and 120 h) after being incubated at 37°C in the dark for 2 h.

### 2.10. Colony Formation Assay

The T24 and 5637 cells (800 cells/well) were seeded into 6-well plates and incubated for 14 d. Then, the cells were fixed with 4% paraformaldehyde and subsequently stained with 1% crystal violet. The number of colonies were then counted.

### 2.11. Wound-Healing Assay

The T24 and 5637 cells were scratched with a 10 *μ*L plastic pipette tip when the cells grew to 90% confluence. The cells were cultured in DMEM medium with 0.5% FBS, and the scratch progression was recorded at 24 h.

### 2.12. Transwell Cell Migration and Invasion Assays

Twenty-four-well Transwell plates with 8 *μ*m pore size (Corning, NY, USA) were used to detect the migratory and invasive ability of the T24 and 5637 cells. For the migration assay, 1 × 10^5^ cells in 100 *μ*L DMEM containing 0.2% FBS and 600 *μ*L of DMEM containing 30% FBS were placed into the upper and lower chambers, respectively. After staining the cells with crystal violet for 15 min, images were taken. For the invasion assay, 500 *μ*L serum-free medium was added to both the upper and lower chambers and incubated in 37°C for 2 h to rehydrate the Matrigel matrix layer. The cells (1 × 10^5^) in 100 *μ*L of DMEM containing 0.2% FBS and 600 *μ*L of DMEM containing 10% FBS were placed into the upper and lower chambers, respectively. After fixing the cells using paraformaldehyde for 30 min and staining with crystal violet for 30 min, images were taken.

### 2.13. Cell Cycle and Apoptosis Assays

The fixed cells were used for cell cycle and apoptosis analyses using flow cytometry (FACS). For cell cycle analysis, the cells were stained with propidium iodide (PI) for 30 min at room temperature. For cell apoptosis assay, the cells were stained with Annexin V-APC for 20 min at room temperature.

### 2.14. Animal Models

T24 (2 × 10^7^) cells were suspended in 200 *μ*L of phosphate-buffered saline (PBS) and then subcutaneously injected into the flanks of BALB/c-nude mice at four weeks of age (eight mice per group). The tumor length and width were measured every 2 d. The tumor volume was calculated using the following equation:

(2) $$\begin{array}{c} \text{tumor volume }\left(\text{m}{\text{m}}^{3}\right)=0.5\times \text{length }\left(\text{mm}\right)\times {\text{width}}^{2}\;\left(\text{m}{\text{m}}^{2}\right). \end{array}$$

The fluorescence of the tumors was observed under a living imager machine before xenograft tumors were resected. Tumor tissues were fixed in 4% phosphate-buffered neutral formalin and subsequently embedded in paraffin for IHC. This study protocol was approved by the Ethics Committee of the First Affiliated Hospital of Bengbu College.

### 2.15. Bioinformatic Analyses

The Tumor IMmune Estimation Resource 2.0 (TIMER2.0; http://timer.cistrome.org/) web server is a comprehensive resource for the systematical analysis of immune infiltrates across diverse cancer types [9]. We explored the association between expression and immune infiltration based on CIBERSORT algorithms.

The Tumor Immune Dysfunction and Exclusion (TIDE; https://tide.dfci.harvard.edu) is a computational framework developed to evaluate the potential of tumor immune escape from the gene expression profiles of cancer samples [10].We demonstrated the relationship of target gene and T cell dysfunction and potential regulators of tumor immune escape through the TIDE website.

### 2.16. Statistical Analysis

All data were shown as means and standard deviation (mean ± SD). SPSS 21.0 (IBM SPSS software, NY, USA) and Prism 7 (GraphPad Software, La Jolla, CA, USA) were used for data analysis. Except for the western blot analysis, all experiments were repeated thrice. Kaplan–Meier analysis was employed for survival analysis. Paired Student's *t*-test was used for the analysis of data obtained from the experiments performed on BC cells and nude mice. A *P* value < 0.05 was regarded as statistically significant.

## 3. Results

### 3.1. MAP9 Was Downregulated after CENPU Knockout and Affected Cell Proliferation

MAP9, the strongest proliferation inhibition target, was screened using qRT-PCR and HCS.

**Figure 1:**
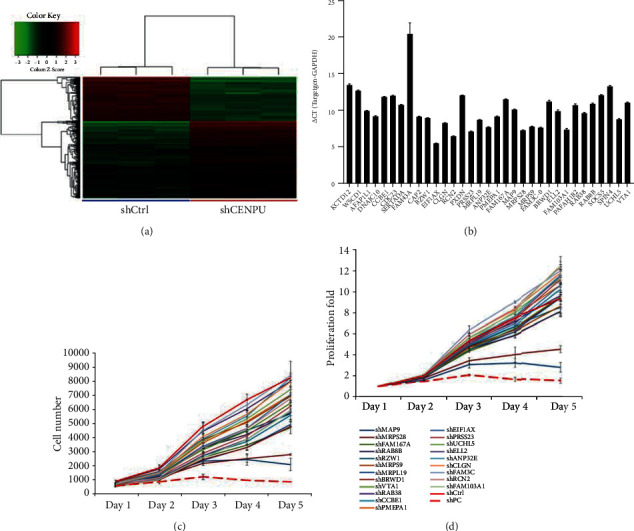
MAP9, the strongest proliferation inhibition target, screened by qRT-PCR and HCS. (a) Clustering of differentially expressed gene sets [2]. (b) qRT-PCR to identify the expression levels of 33 few reported genes selected from 809 downregulated genes. (c) Cell number count of HCS proliferation detection. (d) Proliferation fold of HCS proliferation detection.

Based on our previously reported gene chip results and the gene expression profile analysis results after CENPU knockout, a total of 1274 differentially expressed genes were found, including 809 downregulated genes and 465 upregulated genes [2] (Figure 1(a)). As shown in Figure 1(b), we selected 33 from 809 downregulated genes, which are relatively few reported BC genes. Twenty-one candidate genes with higher expression levels were screened through qPCR quantification for HCS proliferation detection. We obtained two genes with significant proliferation inhibition, by knockdown MAP9 and MRPS28. We selected MAP9 with the strongest proliferation inhibition with a 3.32-fold change as the target of downstream research (Figures 1(c) and 1(d)).

### 3.2. MAP9 Expression Increased in BC and Was a Negative Factor for Prognosis

**Figure 2:**
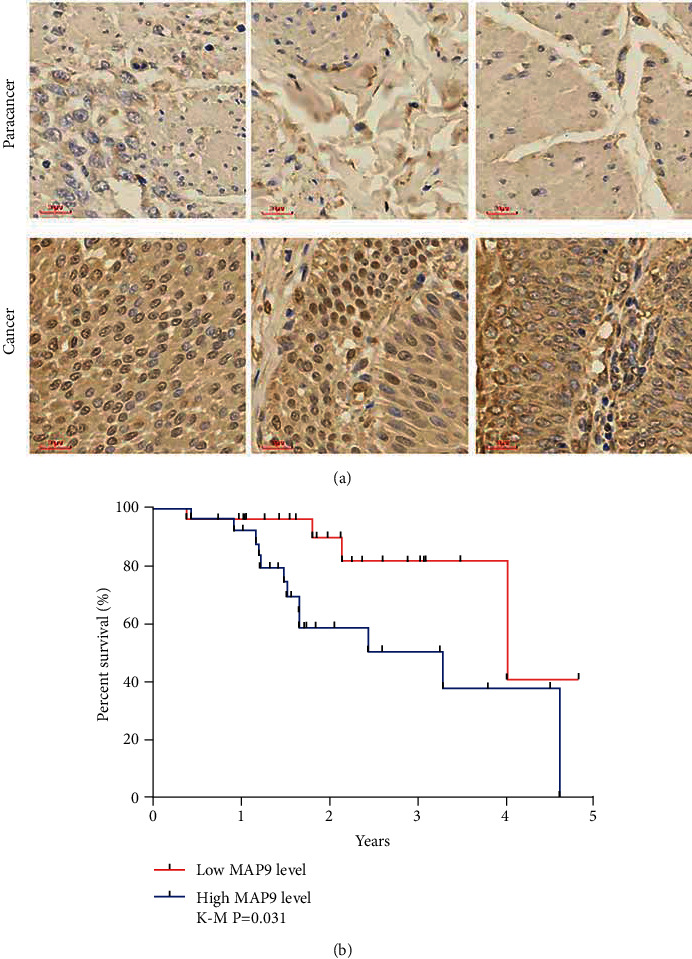
MAP9 expression increased in bladder cancer (BC) cells and was a negative factor for prognosis. (a) MAP9 protein levels in 50 paired BC and paracancerous tissues. (b) The overall survival rate of BC patients with higher and lower MAP9 expression levels. Data are presented as the mean ± standard deviation (SD), and Kaplan–Meier analysis was employed for survival analysis. ^∗^*P* < 0.05, ^∗∗^*P* < 0.01, and ^∗∗∗^*P* < 0.001.

To analyze the expressive level of MAP9 in BC, we performed IHC on tissues obtained from BC patients. As shown in Figure 2(a), the enhanced MAP9 level was detected in cancerous tissues compared to paracancerous tissues. Further, we analyzed the correlation between MAP9 and the prognosis of BC patients. Our results indicated that patients with a lower MAP9 level exhibited a significantly higher, five-year overall survival rate compared to those with a higher MAP9 expression **(**Figure 2(b)**)**.

### 3.3. MAP9 Promoted Cell Proliferation and Inhibited Cell Apoptosis *In Vitro*

**Figure 3:**
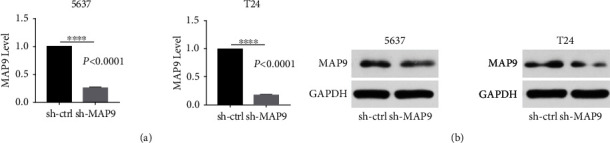
MAP9 expression in the sh-ctrl and short hairpin ribonucleic acid- (RNA-) MAP9 (sh-MAP9) MAP9 cells. (a) Quantitative reverse transcription polymerase chain reaction (qRT-PCR) was conducted to measure the MAP9 messenger ribonucleic acids (mRNAs) in the sh-ctrl and sh-MAP9 cells. (b) Western blotting was conducted to measure the MAP9 proteins in the sh-ctrl and sh-MAP9 cells. Data are presented as the mean ± standard deviation (SD), and paired Student's *t*-test was employed for data analysis. ^∗^*P* < 0.05, ^∗∗^*P* < 0.01, and ^∗∗∗^*P* < 0.001.

**Figure 4:**
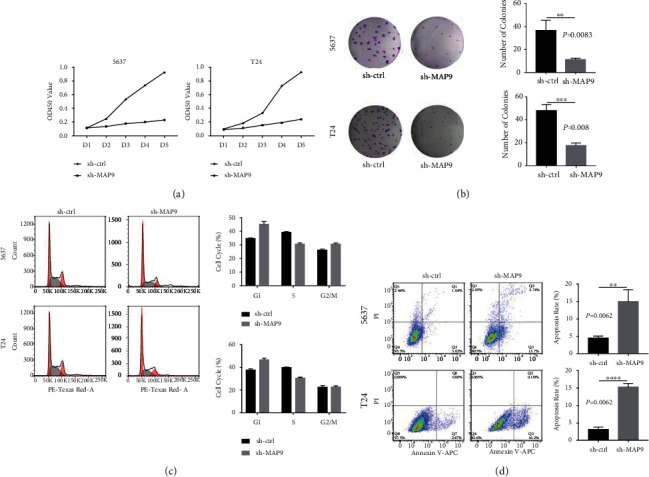
MAP9 promotes cell proliferation and inhibits cell apoptosis *in vitro*. (a) Cell Counting Kit-8 (CCK-8) assay was conducted to analyze the proliferation of T24 and 5637 cells after silencing MAP9. (b) Cell colonies stained with crystal violet were captured and counted after silencing MAP9 in T24 and 5637 cells. (c) Effects of MAP9 silencing on the cell cycle distribution of T24 and 5637 cells. (d) Effects of MAP9 silencing on the cell apoptosis of T24 and 5637 cells. Data are presented as the mean ± standard deviation (SD), and paired Student's *t*-test was employed for data analysis. ^∗^*P* < 0.05, ^∗∗^*P* < 0.01, and ^∗∗∗^*P* < 0.001.

We stably silenced the MAP9 expression in T24 and 5637 cells and divided the cells into sh-ctrl and sh-MAP9 groups (Supplementary Figure 1). The results of the qRT-PCR and western blot analysis demonstrated that MAP9 expression at both the messenger RNA (mRNA) and protein was definitely knocked down in both cells **(**Figure 3**)**. To clarify whether MAP9 is functionally associated with BC growth, we compared the abilities of proliferation and apoptosis between the sh-ctrl and sh-MAP9 cells through a variety of in vitro assays. The results of the CCK-8 assays indicated that downregulation of MAP9 obviously suppressed the proliferation of T24 and 5637 cells (Figure 4(a)). Colony formation assays also confirmed the inhibitory effect of MAP9 silencing on cell proliferation (Figure 4(b)). Further, we explored the cell cycle distribution in the sh-ctrl and sh-MAP9 groups with flow cytometry. An increased number of cells at the growth 1 (G1) phase and a decreased number of cells at the synthesis (S) phase were quantified in the sh-MAP9 cells, whereas a similar number of cells at the growth 2 (G2) phase in sh-the ctrl and sh-MAP9 cells were detected (Figure 4(c)). Flow cytometry analysis based on Annexin V-APC/PI staining illustrated that cell apoptosis was enhanced after silencing MAP9 in both the T24 and 5637 cells (Figure 4(d)). Overall, these results highlighted that MAP9 could promote cell proliferation and inhibit cell apoptosis in T24 and 5637 cells.

### 3.4. MAP9 Promoted Tumor Growth *In Vivo*

**Figure 5:**
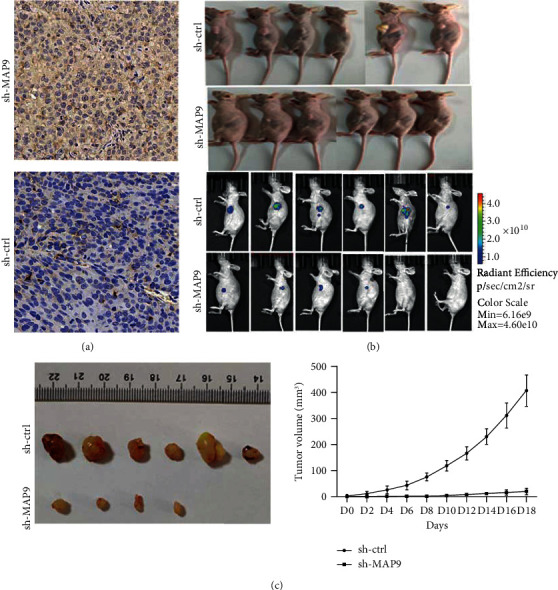
MAP9 promotes tumor growth in vivo. (a) Immunohistochemistry (IHC) staining of the T24 xenograft tumors. (b) *In vivo* imaging technology to monitor the growth of T24 xenograft tumors. Xenograft tumors were generated by injecting sh-ctrl or sh-MAP9 T24 cells. (c) MAP9 knockdown increased the growth of T24 xenograft tumors. Xenograft tumors were generated by injecting sh-ctrl or short hairpin ribonucleic acid- (RNA-) MAP9 (sh-MAP9) T24 cells. The growth of the xenograft tumors was determined by volume. Data are presented as the mean ± standard deviation (SD), and paired Student's *t*-test was employed for data analysis. ^∗^*P* < 0.05, ^∗∗^*P* < 0.01, and ^∗∗∗^*P* < 0.001.

T24 cells with stable knockdown of MAP9 were implanted into nude mice to verify the above results obtained *in vitro*. Immunohistochemistry detected a significant lower MAP9 expression in tumor tissues in the sh-MAP9 group compared to the sh-ctrl group (Figure 5(a)). The fluorescence of tumors observed demonstrated that tumors derived from sh-ctrl cells grow faster compared to those from sh-MAP9 cells (Figure 5(b)). Also, the average tumor volume was uncommonly higher in mice inoculated with sh-ctrl T24 cells (Figure 5(c)) than in the other mice. In conclusion, MAP9 could promote BC growth.

### 3.5. MAP9 Promoted Cell Migration and Invasion *In Vitro*

**Figure 6:**
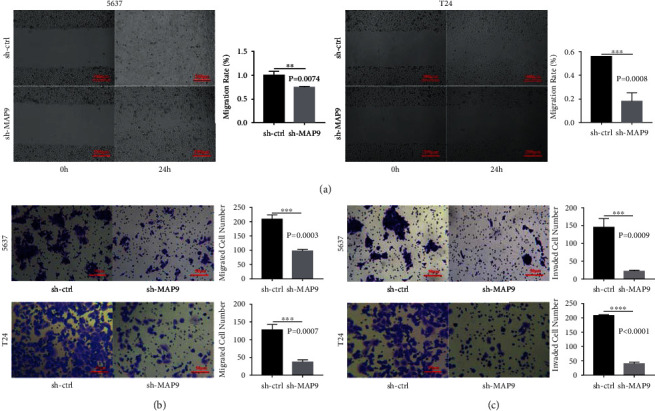
MAP9 promotes cell migration and invasion *in vitro*. (a) The effect of MAP9 silencing on cell migration was analyzed by wound-healing assay. (b) The effect of MAP9 silencing on cell migration was analyzed by Transwell migration assay. (c) The effect of MAP9 silencing on cell invasion was analyzed by Transwell invasion assay. Data are presented as the mean ± standard deviation (SD), and paired Student's *t*-test was employed for data analysis. ^∗^*P* < 0.05, ^∗∗^*P* < 0.01, and ^∗∗∗^*P* < 0.001.

To clarify whether MAP9 is functionally associated with BC metastasis, we compared the ability of migration and invasion between the sh-ctrl and sh-MAP9 cells. The results of the wound-healing migration assay indicated that both T24 and 5637 cells in the sh-MAP9 group displayed a delayed wound healing compared to those in the sh-ctrl group (Figure 6(a)). Also, the Transwell migration assay illustrated that cell migration was strongly damaged after silencing MAP9 in the T24 and 5637 cells (Figure 6(b)). Further, the Transwell invasion assay demonstrated that cell invasion was weaker in the sh-MAP9 group compared to the sh-ctrl group (Figure 6(c)). Taken together, MAP9 could promote BC cell migration and invasion.

### 3.6. MAP9 Promoted BC Growth through G1/S Phase-Related Proteins

**Figure 7:**
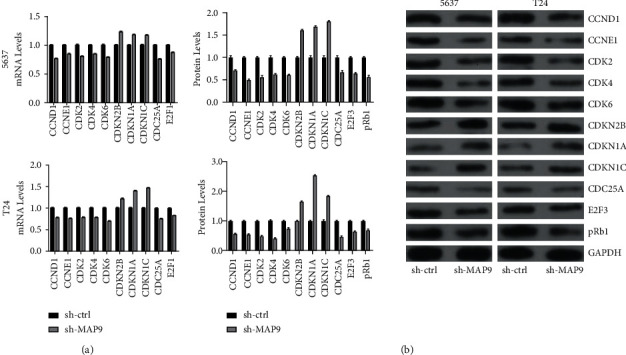
MAP9 regulates growth 1/synthesis (G1/S) transition-related genes. (a) Quantitative reverse transcription polymerase chain reaction (qRT-PCR) was conducted to measure the levels of the genes *cyclin D1*, *cyclin E1*, cyclin-dependent kinase 2 (CDK2), *CDK4*, *CDK6*, *CDK inhibitor 2B* (*CDKN2B*; also named *p15*), *CDKN1A* (also named *p21*), *CDKN1C* (also named *p57*), *cell division cycle 25 A* (*CDC25A*; also named p16), *retinoblastoma transcriptional corepressor 1* (*Rb1*), and *E2F transcription factor* (*E2F1*) in the sh-ctrl (control group) and short hairpin ribonucleic acid- (RNA-) MAP9 (sh-MAP9) MAP9 cells. (b) Western blotting was conducted to measure cyclin D1, cyclin E1, CDK2, CDK4, CDK6, CDKN2B, CDKN1A, CDKN1C, CDC25A, E2F1, and pRb1 protein levels in the sh-ctrl and sh-MAP9 T24 cells. Data are presented as the mean ± standard deviation (SD), and paired Student's *t*-test was employed for data analysis. ^∗^*P* < 0.05, ^∗∗^*P* < 0.01, and ^∗∗∗^*P* < 0.001.

As described above, MAP9 silencing induced the G1 arrest of BC cells, so we detected the expression of G1/S phase-related cell cycle-genes, including *cyclin D1*, *cyclin E1*, cyclin-dependent kinase 2 (CDK2), *CDK4*, *CDK6*, *CDK inhibitor 2B* (*CDKN2B*; also named *p15*), *CDKN1A* (also named *p21*), *CDKN1C* (also named *p57*), *cell division cycle 25 A* (*CDC25A*; also named p16), *retinoblastoma transcriptional corepressor 1* (*Rb1*), and *E2F transcription factor* (*E2F1*) in BC cells with or without MAP9 silencing. The qRT-PCR results indicated that the mRNA levels of *cyclin D1*, *cyclin E1*, *CDK2*, *CDK4*, *CDK6*, *CDC25A*, and *E2F1* decreased in the sh-MAP9 group, and those of *CDKN2B*, *CDKN1A*, and *CDKN1C* increased in the sh-MAP9 group (Figure 7(a)). The results of the western blot analysis confirmed the above results. In detail, the protein levels of cyclin D1, cyclin E1, CDK2, CDK4, CDK6, E2F3, and pRb1 decreased in the sh-MAP9 group, and those of CDKN2B, CDKN1A, CDKN1C, and CDC25A increased in the sh-MAP9 group (Figure 7(b)). Taken together, MAP9 promoted BC growth through regulating G1/S phase-related proteins.

### 3.7. MAP9 Promoted BC Metastasis through Enhancing Epithelial-Mesenchymal Transition (EMT)

**Figure 8:**
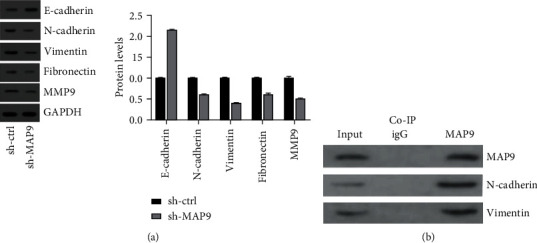
MAP9 modulates EMT-related genes. (a) Western blotting was conducted to measure the levels of E-cadherin, N-cadherin, vimentin, and fibronectin proteins in the sh-ctrl and short hairpin ribonucleic acid- (RNA-) MAP9 (sh-MAP9) MAP9 cells. (b) Coimmunoprecipitation (Co-IP) assay was used to analyze the interaction between MAP9 and E-cadherin, N-cadherin, vimentin, and fibronectin. Data are presented as the mean ± standard deviation (SD), and paired Student's *t*-test was employed for data analysis. ^∗^*P* < 0.05, ^∗∗^*P* < 0.01, and ^∗∗∗^*P* < 0.001.

Epithelial-mesenchymal transition is a key characteristic of metastasis. Here, we explored the effect of MAP9 silencing on EMT-related markers. We found that the protein level of E-cadherin increased in the sh-MAP9 T24 cells, whereas the protein levels of N-cadherin, vimentin, and fibronectin decreased in the sh-MAP9 T24 cells (Figure 8(a)). Further, we conducted a Co-IP assay to analyze the interaction between MAP9 and N-cadherin and vimentin. The results of Co-IP suggested that MAP9 could bind to N-cadherin and vimentin (Figure 8(b)). Overall, MAP9 promoted BC metastasis through promoting the EMT process.

### 3.8. MAP9 Regulated Cell Cycle-Related Genes and EMT via Transforming Growth Factor Beta 1 (TGF-*β*1)

**Figure 9:**
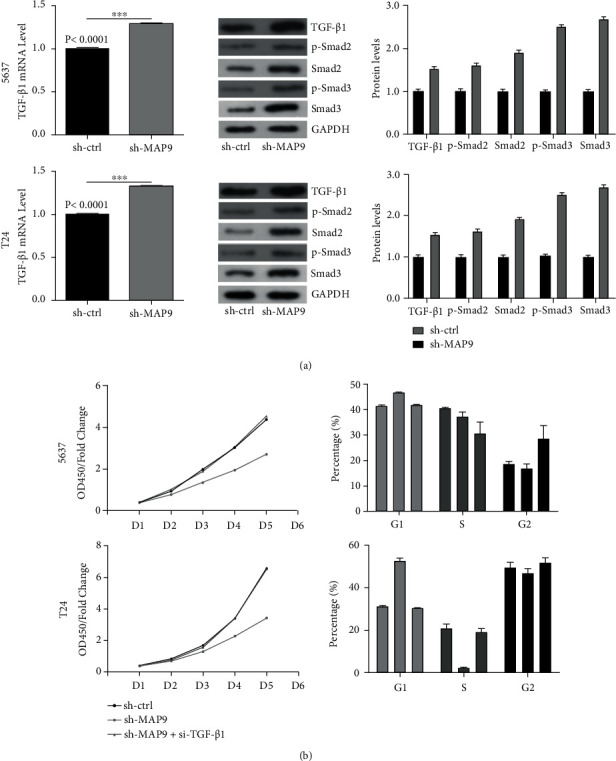
MAP9 regulated cell cycle-related genes and epithelial mesenchymal transition (EMT) via the transforming growth factor beta 1 (TGF-*β*1) pathway. (a) The messenger ribonucleic acid (mRNA), protein levels of TGF-*β*1, and phosphorylated mothers against decapentaplegic homolog (p-SMAD)2 and p-SMAD3 levels increased in the short hairpin ribonucleic acid- (RNA-) MAP9 (sh-MAP9) MAP9 group compared to the negative control (NC) group. Also, significantly enhanced phosphorylation levels of SMAD2 and SMAD3 were detected in the sh-MAP9 cells. (b) The proliferation ability of both 5637 and T24 cells, detected by Cell Counting Kit-8 (CCK-8) assay and flow cytometry assays, was rescued. Data are presented as the mean ± standard deviation (SD), and paired Student's *t*-test was employed for data analysis. ^∗^*P* < 0.05, ^∗∗^*P* < 0.01, and ^∗∗∗^*P* < 0.001.

The TGF-*β*1 pathway is one of the main pathways that plays a role in the growth, differentiation, and metastasis of BC. After TGF-*β*1 binds to its receptor, mothers against decapentaplegic homolog (SMAD)2 and SMAD3 are phosphorylated, and the downstream targets, such as EMT markers and cell cycle genes, are subsequently activated. As shown in Figure 9(a), both the mRNA and protein levels of TGF-*β*1 increased in the sh-MAP9 group compared to the NC group. Also, significantly enhanced phosphorylation levels of SMAD2 and SMAD3 were detected in the sh-MAP9 cells. Further, we silenced TGF-*β*1 in the sh-MAP9 BC cells and found that the proliferation ability of both 5637 and T24 cells detected by CCK-8 and flow cytometry assays was rescued (Figure 9(b)). These results indicated that downregulation of MAP9 expression levels may activate the TGF-*β*1 pathway by switching on the SMAD-dependent signaling pathway.

### 3.9. MAP9 Modulates Immune Escape of BC

**Figure 10:**
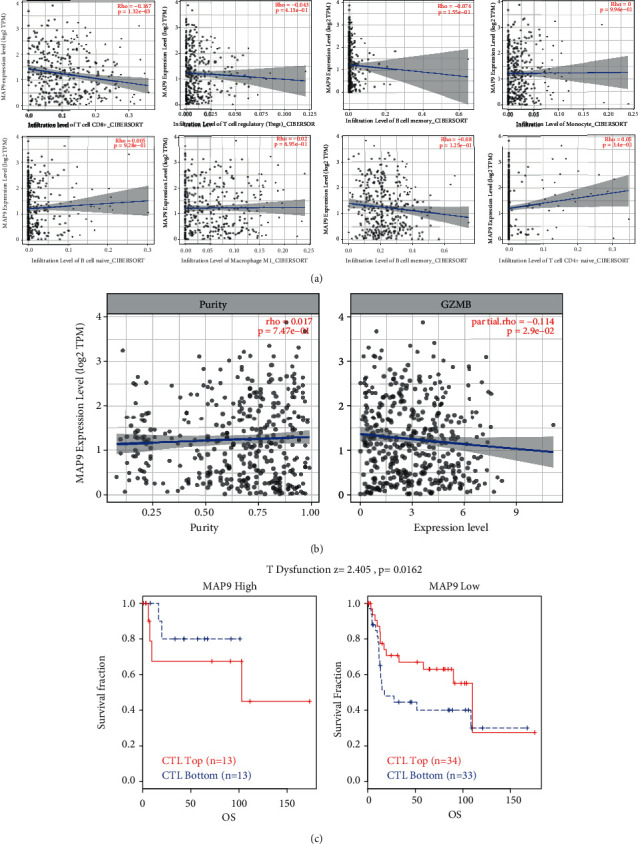
MAP9 mediates immune escape in bladder cancer. (a) The correlation between MAP9 and immune cell infiltration in the TCGA-BLCA cohort. (b) The correlation between MAP9 and GZMB in the TCGA-BLCA cohort.(c) The differences of prognosis between different expressed MAP9 and cytotoxic T lymphocyte infiltration with TIDE algorithm in the GSE31684 cohort.

As a critical regulator of the inflammatory response, TGF-*β*1 is essential for the formation of immune tolerance. TGF-*β*1 acts as a “gatekeeper” of the tumor microenvironment and is becoming one of the most promising targets in cancer immunotherapy [11]. However, the role of MAP9 in tumor immunity is remains unknown. So we employed TIMER2.0 to explore that MAP9 was negatively correlated with CD8+ T cell infiltration in the TCGA-BLCA cohort(Figure 10(a)). And there was inverse correlation between MAP9 and the cytotoxic T cell marker granzyme B after purity adjustment (Figure 10(b)). Then, we analyzed the bladder cancer cohort GSE31684 with the TIDE algorithm. The T cell dysfunction score of MAP9 was positive, and patients with high MAP9 expression had worse prognosis with high cytotoxic T lymphocyte infiltration, whereas patients with low MAP9 expression had the opposite prognosis (Figure 10(c)).

## 4. Discussion

MAP9 is a mitosis-associated protein localized in the microtubules in the interphase, which is related to bipolar spindle assembly, centrosome regulation, and cytokinesis during mitosis [3]. The loss of MAP9 induced abnormal spindles with chromosome congression and segregation defects and severe mitotic defects with delayed mitotic progression [3, 12]. During cell mitosis, MAP9 is phosphorylated by polo-like kinase 1 (Plk1) and Aurora kinase A (AURKA) [3, 12]. The abnormal expression of Plk1 and AURKA is proven to be toughly related to the tumorigenesis of many cancer types [13–15]. Considering the interactions of MAP9 with Plk1 and AURKA, we hypothesized that MAP9 is vital in tumor progression. Actually, previous studies did uncover that MAP9 is associated with the progression of gastric cancer [16], colorectal cancer [7, 17], and HCC [6, 8]. However, the specific role of MAP9 in BC is still unknown.

Abnormal expression and methylation have been observed in colorectal cancer and HCC. In colorectal cancer, the MAP9 expression was frequently silenced, and MAP9 loss initiated tumorigenesis and was associated with poor survival of patients [7, 17]. In HCC, a decreased MAP9 mediated by its promoter hypermethylation had been measured, and MAP9 downregulation or promoter hypermethylation was related to recurrence and poor survival [6, 8]. These studies indicated that MAP9 expression was reduced in tumors and was a disease marker for tumors. Combining our HCS results, we hypothesized that MAP9 expression in BC is disordered and is related to the prognosis of BC patients. To verify our hypothesis, we detected MAP9 levels in BC through IHC, and we found an increased MAP9 expression in cancerous samples. We further divided all BC patients included in the study into two groups based on the expression of MAP9 and compared the clinical parameters and overall survival rates in the two groups. We found that MAP9 expression was positively related to the tumor size and negatively related to the overall survival rates of patients. These results indicated that MAP9 might promote tumor growth in BC.

To determine whether MAP9 could promote tumor growth, we compared the proliferative abilities of BC cell lines and tumor size in nude mice with or without MAP9 silencing. We found that knocking down MAP9 attenuated cell proliferation via inducing the G1/S cell cycle arrest and inhibited tumor growth. The G1/S transition is mediated by a complicated process. In detail, cyclin D binds to CDK4 and CDK6 to form a complex, and the complex can be activated by factors, such as CDC25A, or be blocked by CDK inhibitors, such as CDKN2B, CDKN1A, and CDKN1C [18–22]. Because MAP9 regulates G1/S transition in BC, we detected the expression of related genes in BC cells with or without MAP9 loss. We found that cyclin D1, cyclin E1, CDK2, CDK4, CDK6, CDC25A, and E2F3 were positively regulated by MAP9, whereas CDKN2B, CDKN1A, and CDKN1C were negatively regulated by MAP9. These results indicated that G1/S transition-related genes are key factors causing MAP9 to promote BC cell proliferation.

Although none of the patients in this study experienced lymph node or distant metastasis, metastasis is an outstanding feature of tumors and largely affects patients' prognosis. Therefore, we also explored the correlation between MAP9 expression and BC metastasis. We found that less cells migrated and invaded after MAP9 silencing. Epithelial mesenchymal transition is a classic process that is involved in tumor metastasis. Thus, we compared the levels of EMT-related genes in the sh-ctrl and sh-MAP9 cells. We found that E-cadherin was negatively affected by MAP9, whereas N-cadherin, vimentin, and fibronectin were positively affected by MAP9. Taken together, we concluded that MAP9 enhances BC metastasis by promoting the EMT process.

The TGF-*β*1 signaling pathway widely participates in tumor progression and is closely related to the EMT and tumor immune process. The process of TGF-*β*1 activation is as follows: TGF-*β*1 firstly binds to its membrane ligand TGF-*β* receptor type-2 (T*β*RII), and then, T*β*RII binds to and phosphorylates TGF-*β* receptor type-1 (T*β*RI). Phosphorylated T*β*RI leads to the phosphorylation of SMAD2 and SMAD3, and phosphorylated SMAD2 and SMAD3 transduce the signal into the nucleus. The SMAD signaling pathway can inhibit the expression of cellular Myc (c-Myc) and promote the expression of cell cycle inhibitory proteins P21 and P15, leading to cell cycle arrest. This pathway enables TGF-*β*1 to inhibit cell proliferation [23–25], which was confirmed through our research findings. We found that the TGF-*β*1 level and phosphorylation levels of SMAD2 and SMAD3 were positively regulated by MAP9 in BC cells. Based on previous studies and what we have obtained in this study, we suggested that MAP9 mediates the TGF-*β*1 signaling pathway and subsequently activates G1/S transition genes and enhances the EMT process. MAP9 is negatively correlated with CD8+ T cell infiltration in BC patients and promotes T cell dysfunction, resulting in immune escape. MAP9 has a significant influence on tumor immune microenvironment and could be a potential biomarker for immunotherapy.

Although this study revealed the role of MAP9 in BC partly, limitations still existed. Firstly, we only used one cell line (T24) to conduct *in vivo* experiments and to study the mechanism of MAP9. Secondly, TGF-*β*1 is a secreted protein, which makes it difficult to explore how MAP9 upregulates TGF-*β*1. As a result, we failed to explore the mechanism of MAP9 in upregulating TGF-*β*1.

In conclusion, we screened and found MAP9 as a potential target for the treatment of human BC by mediating the TGF-*β*1 signaling pathway and subsequently activating G1/S transition genes and enhancing the EMT process to promote BC growth and metastasis. And MAP9 also plays an important role in the immune escape of BC patients. Our study provides a comprehensive analysis of MAP9 and revealed MAP9 as a potential target for gene therapy of human bladder cancer.

## 5. Conclusion

In the current research, we found that MAP9 can promote BC progression and immune escape activity through the TGF-*β*1 pathway and is a potential novel target for therapies of BC.

## Data Availability

The data and materials used to support the findings of this study are available from the corresponding author upon request.

## Abbreviations

MAP9: Microtubule-associated protein 9
BC: Bladder cancer
qRT-PCR: Quantitative reverse transcription polymerase chain reaction
Co-IP: Coimmunoprecipitation
EMT: Epithelial mesenchymal transition
RIPA: Radioimmunoprecipitation assay
ELISA: Enzyme-linked immunosorbent assay.

## Supplementary Materials

Supplementary Materials Table S1: primers used in this study. Table S2: antibodies used in this study. Figure S1: green fluorescent protein expression in the sh-ctrl and sh-MAP9 cells.
