# Supplementary material for: MAP9 Exhibits Protumor Activities and Immune Escape toward Bladder Cancer by Mediating TGF-β1 Pathway
Source: J Oncol. 2022 May 24;2022:3778623. doi: 10.1155/2022/3778623 (PMC9155934; doi:10.1155/2022/3778623)
Supplement: Supplementary Materials — Table S1: primers used in this study. Table S2: antibodies used in this study. Figure S1: green fluorescent protein expression in the sh-ctrl and sh-MAP9 cells. [file 3778623.f1.docx]

**Supplemental tables**

| Gene | Forward (5’-3’) | Reverse (5’-3’) | Product Length |
| --- | --- | --- | --- |
| MAP9 | TGCACCTTCTTCCCTTCCAAC | TTTTCCAGATGCGTTTCCCTC | 175 |
| E2F1 | CTCCTCGCAGATCGTCATCA | AAGCGCTTGGTGGTCAGATT | 330 |
| CDK6 | AAGTCTTGCTCCAGTCCAGC | CTGGGAGTCCAATCACGTCC | 150 |
| CDC25A | TTCCTCTTTTTACACCCCAGTCA | TCGGTTGTCAAGGTTTGTAGTTC | 173 |
| CDKN1A | CGATGGAACTTCGACTTTGTCA | GCACAAGGGTACAAGACAGTG | 220 |
| CDKN1C | GCGGCGATCAAGAAGCTGT | GCTTGGCGAAGAAATCGGAGA | 52 |
| CCNE1 | ACTCAACGTGCAAGCCTCG | GCTCAAGAAAGTGCTGATCCC | 141 |
| TGFB1 | CAATTCCTGGCGATACCTCAG | GCACAACTCCGGTGACATCAA | 86 |
| CDK2 | GTACCTCCCCTGGATGAAGAT | CGAAATCCGCTTGTTAGGGTC | 75 |
| CDK4 | CTGGTGTTTGAGCATGTAGACC | GATCCTTGATCGTTTCGGCTG | 88 |
| CCND1 | CAATGACCCCGCACGATTTC | CATGGAGGGCGGATTGGAA | 146 |
| CDKN2B | CGGGGACTAGTGGAGAAGGTG | CCATCATCATGACCTGGATCGC | 100 |
| GAPDH | TGACTTCAACAGCGACACCCA | CACCCTGTTGCTGTAGCCAAA | 121 |

Table S1: Primers used in this study

| Antigens | Manufacturers | Application |
| --- | --- | --- |
| MAP9 | #26078-1-AP, Proteintech, Rosemont, IL, USA | 1:1000 for WB and CO-IP 1:25 for IHC |
| E2F3 | #ab50917, Abcam, Cambridge, MA, USA | 1:1000 for WB |
| CDK2 | #ab32147, Abcam, Cambridge, MA, USA | 1:1000 for WB |
| CDK4 | #ab32147, Abcam, Cambridge, MA, USA | 1:500 for WB |
| CDK6 | #ab151247, Abcam, Cambridge, MA, USA | 1:800 for WB |
| CCND1 | #ab16663, Abcam, Cambridge, MA, USA | 1:200 for WB |
| CCNE1 | #20808, Cell Signaling Technology, Beverly, MA, USA | 1:1000 for WB |
| CDKN2B | #AF6474, Beyotime, Shanghai, China | 1:2000 for WB |
| CDKN1A | #orb48324, Biorbyt, Cambridge, UK | 1:1000 for WB |
| CDKN1C | #ab75974, Abcam, Cambridge, MA, USA | 1:500 for WB |
| CDC25A | #ab47400, Abcam, Cambridge, MA, USA | 1:1000 for WB |
| TGF-β1 | #ab179695, Abcam, Cambridge, MA, USA | 1:1000 for WB |
| Rb1 | #10048-2-lg, Proteintech, Rosemont, IL, USA | 1:2000 for WB |
| p-Smad2 | #3104, Cell Signaling Technology, Beverly, MA, USA | 1:1000 for WB |
| p-Smad3 | #9520, Cell Signaling Technology, Beverly, MA, USA | 1:1000 for WB |
| E-cadherin | #ab133597, Abcam, Cambridge, MA, USA | 1:1000 for WB |
| N-cadherin | #ab76057, Abcam, Cambridge, MA, USA | 1:1000 for WB and CO-IP |
| Vimentin | #ab8978, Abcam, Cambridge, MA, USA | 1:200 for WB and CO-IP |
| Fibronectin | #ab2413, Abcam, Cambridge, MA, USA | 1:500 for WB |
| MMP9 | #ab38898, Abcam, Cambridge, MA, USA | 1:1000 for WB and CO-IP |
| GAPDH | #10494-1-AP, Proteintech, Rosemont, IL, USA | 1:10000 for WB |
| Goat anti-Mouse IgG | #31160, ThermoFisher, Waltham, MA, USA | 1:5000 for WB |
| Goat anti-Rabbit IgG | #31210, ThermoFisher, Waltham, MA, USA | 1:5000 for WB |
| HRP-linked anti-rabbit IgG | #31402, ThermoFisher, Waltham, MA, USA | 1:5000 for IHC |

Table S2: Antibodies used in this study

**Supplementary Figure 1. Green fluorescent protein expression in the sh-ctrl and short hairpin ribonucleic acid (RNA)-MAP9 (sh-MAP9) MAP9 cells.** (**A**) Fluorescence of 5637 cells in the sh-ctrl and sh-MAP9 groups under the microscope. (**B**) Fluorescence of T24 cells in the sh-ctrl and sh-MAP9 groups under the microscope.
